# Transradial vs. Transfemoral Approach in Cardiac Catheterization: A Literature Review

**DOI:** 10.7759/cureus.1309

**Published:** 2017-06-03

**Authors:** Ibrar Anjum, Muhammad Adnan Khan, Muhammad Aadil, Aniqa Faraz, Mudassir Farooqui, Amerah Hashmi

**Affiliations:** 1 Internal medicine, University of health science Lahore; 2 Neurosciences & Psychology, California Institute of Behavioral Neurosciences & Psychology; 3 Department of Psychiatry and Mental Health, Rush University Medical Center; 4 Department of Internal Medicine, King Edward Medical University Lahore, Pakistan; 5 Public Health, University of Oklahoma

**Keywords:** transradial vs. transfemoral approach in catheterization, radial vs. femoral access, percutaneous coronary intervention, transradial and transfemoral approach, catheterization, transradial in coronary heart disease

## Abstract

The main objective of this review paper is to study the comparison between transradial and transfemoral approach in catheterization. Transradial and transfemoral are two main approaches which are used as a diagnostic and therapeutic purpose in catheterization. The transradial approach in interventional cardiology is safe, effective, and feasible as compared to the transfemoral approach. The aim of this study is to compare pros and cons of transradial vs. transfemoral approach in catheterization.

We conducted this systematic review on the role of transradial vs. transfemoral catheterization. The articles included real human data on interventional approaches. Reviews on these strategies were conducted in PubMed, medical literature analysis and retrieval system online (MEDLINE), Cochrane, Medscape and National Institute of Health. To maintain a high standard of review, studies published in all non-famous journals were excluded.

Data collected from the studies have suggested that transradial approach has less bleeding complications, cost effective, decreased hospital mortality rate, and less access site complications as compared to transfemoral approach. However, longer procedural duration and radiation exposure are still concerns regarding transradial approach.

The findings of the present study show that transradial approach in catheterization is safe, effective, and feasible as compared to the transfemoral approach. However, duration and radiation exposure are higher in the transradial access. Several studies suggest that the modern approach overweight in benefits with the comparison to the classical approach.

## Introduction and background

The interventional cardiology deals with the catheter-based treatment of structural heart diseases. It uses diagnosing and treating the cardiovascular diseases, including congenital and structural heart diseases through catheter-based procedures such as angioplasty and stenting. Andreas Gruentzig is considered as the father of interventional cardiology [1]. He was the first who performed successfully coronary angioplasty on an awake human in September 1977. He opened occluded left anterior descending (LAD) which is a branch of left coronary artery usually involved in most myocardial infarction patients. Until the 1950s, Sones techniques introduced by Dr. Mason Sone which is popular to cut down soft tissue to visualized artery or vein to pass the catheter was used. The percutaneous approach that is widely used today was developed by radiologist Sven-Ivar Seldinger [2]. Percutaneous coronary intervention (PCI) also known as angioplasty is used to open blocked coronary vessels in the heart. It improves blood flow and decreases the mortality in acute coronary syndrome patients. Mostly the procedures are performed in cardiology via catheterization by either femoral or radial access.

The main advantages of using catheterization approach are a rapid recovery with early ambulation, less postoperative complications, less hospital cost and less chance of scars formation. Thus, it increases the patient comfort [3]. Additionally, angioplasty is now considered as a gold standard procedure for the treatment of acute myocardial infarction. The purpose of writing this paper is to investigate the pros and cons of transradial vs. transfemoral approach catheterization.

Transfemoral is considered as a classical approach over transradial due to the unlimited repetition of puncturing, easy access, less radiation time, and less contrast. In the last two decades, transradial approach emerged as mostly being used for the interventional and diagnostic approach in cardiology. In 1989 the transradial approach coronary angiography was reported for the first time in Campeau [4]. The reason behind the popularity of transradial approach is reduced bleeding risk, reduced hematoma formation, early discharge, it is patient preferred, low cost, and lower risk of morbidity and mortality [3, 5-7]. Many trials have proved that transradial approach has the lower risk of bleeding in ST-Segment elevation myocardial infarction (STEMI) patients using anticoagulation as compared to transfemoral approach [8-10]. The transition from femoral access to a radial access is safe and efficient in many procedures in interventional cardiology. It has fewer side effects of low bleeding, pseudoaneurysm, low cost, morbidity and mortality [11- 12]. The outcome of the transradial is much better as compared to the transfemoral approach in catheterization [1-4, 6].

## Review

### Method

We conducted this systematic review on the role of transradial versus transfemoral in catheterization. Reviews on these approaches were conducted in PubMed, MEDLINE, Cochrane, Medscape and National Institute of Health. Cross checking of references led to the identification of additional relevant references. The decision to involve or eliminate reviews and data extraction was completed by the authors and any controversy was settled by discussion. The articles included real human data on interventional approaches. Articles related to interventional cardiology were thoroughly searched and later the articles focusing mostly on transradial versus transfemoral approach in these patients were searched. However, the reviews with high possibility of bias or the studies with hazy and confounded data were excluded. To maintain the high standard of review, studies published in all non-famous journals were excluded. The animal studies were also excluded to maintain focus on human heart diseases. We reviewed 220 articles initially and 43 were included based on their relevance to the role of transradial versus transfemoral approach and its pros and cons in interventional cardiology.

### Discussion

Andreas Gruentzig is considered the father of interventional cardiology because he was the first who successfully performed coronary angioplasty in a human patient in September 1977 [13]. The early results of this treatment, despite using only a carefully kitchen built catheter lab were quite good. The patient was angina free after this treatment. The excellent results of this treatment were lead to the rapid acceptance and growing of angioplasty treatment option. A lot of procedures can be performed on the heart by cardiac catheterization. The classical approach is through femoral sheath poking into the femoral artery. The transradial artery may also be used for cannulation due to numerous benefits over transfemoral, like the accessibility of the artery in most patients, fewer chances of hematoma formation even in anticoagulated patients, the patient comfort, because patients are capable of ambulating immediately following the procedure and easily stopping the bleeding by compression.

### Transfemoral approach- the classical approach

The femoral approach technique can be performed repeatedly in the same patient. The femoral artery is easily palpable and allows for easy access. The transfemoral approach is the method of choice in those patients with absent/difficult to palpate radial and brachial pulsations and for those instances, where the transradial approach has been unsuccessful when the large-caliber catheters are used. In 1970s large guide catheters were used in angioplasty which required large lumen arterial access, so transfemoral approach became the main source of arterial access for coronary catheterization and intervention. It has gained universal acceptance because of the extensive usage and the workforce experience and easy access; furthermore, it also enables the use of larger sheaths and other equipment for the evolution of known complications.

Before 2008, the transfemoral approach was considered as the main route of arterial access for cardiac catheterization in the United States. However, transradial cardiac catheterization in the United States is growing with time due to the significant risk of transfemoral associated major and minor vascular complications related to transfemoral approach. The American College of Cardiology defines vascular complications as minor or major. Minor vascular complications were defined as any of the following: hematoma < 10 cm, fistulae, or pseudoaneurysm [14]. However, the major vascular complications were defined as death caused by major vascular bleeding leading to > 3 g fall in hemoglobin level due to retroperitoneal bleeding or administration of blood transfusions or vascular repair, vessel occlusion, or loss of pulse [15].

The most common femoral approach vascular complications are; the access site bleeding, hematoma, arteriovenous (AV) fistula, retroperitoneal bleeding, and pseudoaneurysm. In the United States, the proportion of transradial percutaneous coronary intervention (PCI) increased gradually, from 1.2% in 2007 to 16.1% in 2012 and a total of 6.3% of total procedures from 2007 to 2012 [11]. The complication for this procedure includes bleeding which may sometimes require transfusion to treat the bleeding complication. However, the many studies appoint strongly towards the -evidence that these post-procedural bleeding especially retroperitoneal bleeding is associated with a bad prognosis and the blood transfusion after the procedure is also associated with poor prognosis [12].

Elderly and obese patients are more prone to the risk of bleeding complications after the transfemoral approach. A retrospective cohort study is performed in 21,103 obese patients (BMI 40) who underwent percutaneous coronary intervention (PCI) and angiography. The study reports that the patients with the transfemoral approach had more chances of bleeding and access side vascular complications as compared to the transradial approach patients who had fewer chances of bleeding complications. The patient morbidity rate had reduced with transradial approach [16]. The pros and cons of transfemoral approach in cardiac catheterization have been summarized in the (Table-1) [1-2-3, 12-13-14-15].

**Table 1:** Transfemoral approach’s pros and cons

Pros	Cons
Availability of trained and experienced doctors in this approach Large artery diameter Procedural complications are known and its prevention is also available Better for patients with extensive peripheral arterial disease (PAD) Long history of successful approach	Risk of Bleeding is high Longer hospital stay A Pseudoaneurysm and Clot formation Higher procedural cost Femoral artery is the only source of blood to the leg

### Transradial approach- the modern approach

Transradial catheterization is currently more popular in Asia and Europe. The transradial approach coronary angiography was reported for the first time in Campeau [2] and subsequently for transradial angiography in 1989 [17] and coronary stenting in 1993 [18]. The percutaneous coronary intervention (PCI) via the transradial (TR) approach has gained increasing popularity due to less bleeding complications. In the 1970’s and 80’s, many cardiologists were proficient in transradial approach, thus finishing a catheterization only 10 to 15 minutes longer than transfemoral approach. The radial artery is very superﬁcial, making it easy to puncture and bleeding is controlled by compression. Anatomically, there is no major nerves or veins present near the radial artery, thus, minimizing the risk of nerve and vascular injuries. In the past 10 years, the benefits of transradial access have been documented in many studies. Some benefits of the transradial approach include; less bleeding complications, [1-3, 6, 19-20] lower morbidity, early ambulation, associated with lower total hospital costs compared with transfemoral intervention (TFI) approach [5], patient preference and comfort, easy to compress and hemostasis, same day discharge is possible, less chance of developing ischemia due to dual blood supply of hand and easy access for the patient of myocardial infarction (MI) and aortic aneurysm (AA) [21-22].

Vorobcsuk, et al. performed a pool of data collection on the population of 3324 patients in 12 different studies, who underwent percutaneous coronary intervention [PCI] either via transradial or transfemoral approach. They found a 70% risk reduction in access-site bleeding with the transradial approach. This attainment further converted into the lower incidence of hospital major adverse cardiac events and mortality [23].

Studies have suggested that transradial approach may reduce hospital mortality among patients with STEMI. The study has shown that 294,769 patients undergone PCI for ST-segment elevation myocardial infarction [STEMI] in between 2007 and 2011. Data shows less bleeding complications and lower hospital morbidity and mortality rate by transradial approach [24]. Radial approach is considered better for coronary stenting than femoral in patients with the acute coronary syndrome. Access site bleeding complications are less and shorter hospital stay results in decrease morbidity and mortality [19]. A significant benefit of transradial catheterization is faster, more comfortable recovery. A cohort study included 334 end stage liver failure patients, have shown that transradial approach decreased the risk of bleeding, lower vascular complications and pseudoaneurysm as compared to the classical approach in patients with end-stage liver disease [25].

In some instances, the patients with ST-elevation myocardial infarction [STEMI] may require antithrombotic therapy and have the high risk of bleeding. However, the studies have shown that transradial approach is safe and efficient for coronary angiography in these patients [26]. A study conducted by Sciahbasi AT showed the frequency of bleeding and mortality due to transradial approach and has been demonstrated in an extensive study in PCI [27]. Similar observational study on thousand non-ST-segment elevations myocardial infarction [NSTEMI] patients has been demonstrated in transradial treated cases [28].

Greenberg G, et al. performed analysis on 4873 consecutive patients from April 2007 to July 2012 who underwent PCI at a community hospital. He studied a comparison between transradial versus transfemoral approaches in these patients. The study showed that the hospitalization was shorter in the transradial intervention as compared to transfemoral intervention [29]. PCI is still relatively underused in the United States and the reason behind is the immense challenge in various aspects, including slight diameter calcification and fibrosis in senior patients. The small size and the incidence of radial artery spasm create problems in obtaining vascular access. Radial artery occlusion is another major complication of transradial approach, but most of them are asymptomatic. Radial artery occlusion can be reduced by using small diameter catheter, using anticoagulation and applying enough pressure on the radial artery to stop bleeding and by this way we can reduce asymptomatic occlusion significantly [30].

Coronary angiography via transradial approach become very popular worldwide and is becoming more accepted in recent years, based on a reduction in vascular complications and mortality as compared with the transfemoral approach [31-32]. However, these benefits come at the cost of increased procedure time and fluoroscopy dose [33-34]. There is also a concern that transradial approach may delay time to reperfusion if vascular access time is increased or difficult anatomy requiring multiple catheter exchanges. However, because of its realistic and feasible approach, the United States will continue to experience a shift towards a transradial approach. Sooner rather than later, the landscape in the United States will mirror that of Europe and Asia [35-39]. In these days, both patients and staff prefer the transradial approach because of these benefits. As a result of these advantages, transradial approach is popular in worldwide. The pros and cons of transradial approach in cardiac catheterization have been summarized in the (Table-2) [1-2-3, 19-20-21-22-23].

**Table 2:** Transradial approach’s pros and cons

Pros	Cons
Low morbidity and mortality Risk of bleeding complication and hematoma formation are low Low procedural cost Early discharge even the same day Radial artery is not the only source of blood to the hand No nothing by mouth (NPO) restriction soon after the procedure	Unavailability many trained and experienced doctors Due to small radial artery diameter, procedure is difficult to perform More time is required as compared to femoral approach Procedural complications such as shunt or fistula Post-procedural severe vascular spasm like (Raynaud’s)

In the last decade, transradial and transfemoral approaches in the cardiac catheterization have been largely studied and have been an area of major interest in intervention cardiology [2, 5, 13, 25, 35, 37]. Though, the data on both approached implies on their efficacy, practicality, and benefits; however, both approaches also have their complications. Through the available data in the scientific literature, the findings from some of the relevant studies showing the role of transradial and transfemoral approaches in the cardiac catheterization have been summarized in the (Table - 3) [16, 19, 24-25, 40-41-42-43].

**Table 3:** The role of transradial and transfemoral approaches in the cardiac catheterization in relevant studies

Author and the year of publication	Study design	Sample size	Diagnostic criteria	Study findings
Kedev S, et al. 2014 [3]	Clinical trial	STEMI patients (n=1808) who underwent PCI using transradial approach (n=1162) and transfemoral approach (n=646) from October 2007 to December 2010 were enrolled	Comparison of short- and long-term outcomes of transradial approach (TRA) versus transfemoral approach (TFA) for primary percutaneous coronary intervention (PPCI)	Complete transition from femoral access to a radial access is safe and effective for STEMI patients undergoing PPCI, with a favorable effect on short- and long-term outcomes
Roussanov O, et al. 2007 [5]	Cohort study	from October 2004 to May 2006, a total of 181 patients who underwent diagnostic cardiac catheterization at Salem Veterans Affairs Medical Center	Cost comparisons have been made between the radial and femoral approaches to diagnostic cardiac catheterization	The radial artery approach to diagnostic cardiac catheterization is clearly more cost effective than the femoral approach.
Jang JS, et al. 2012 [10]	Systematic review and meta-analysis	Twenty-one studies involving 8,534 patients were identified	TR approach is associated with lower incidence of complications in vascular access site and improved clinical outcomes compared with TF approach in the setting of STEMI	TR – PCI reduces the risk of significant periprocedural bleeding and improve clinical outcomes in patients with STEMI
Tewari S, et al. 2013 [15]	Clinical trial	26,238 patients, who underwent PCI procedures 55.65% and 44.35% procedures were done through TF and TR approach	Comparison of transradial and transfemoral artery approach for percutaneous coronary procedures	Number of TR approaches have increased significantly with reduced complication rates and comparable success rate to TF approach, with the additional benefits in terms of patient comfort, preference and reduced cost of procedure
Hibbert B, et al. 2012 [16]	Cohort study	Out of 21,103 patients procedures, 564 were performed in unique EO patients: 203 via the transradial approach and 361 via the transfemoral approach	Transradial versus transfemoral access for coronary angiography and PCI in patients with a body mass index ≥ 40	TF access for coronary angiography and PCI was associated with more access site and bleeding complications compared with a TR approach
Mann T, et al. 1998 [19]	Prospective randomized clinical trial	142 patients	To compare the transradial approach with the transfemoral approach for coronary stenting in patients with acute coronary syndromes	Coronary stenting from the transradial approach is efﬁcacious in patients with acute coronary syndromes
Baklanov DV, et al. 2013 [24]	Clinical trial	294,769 patients undergoing PCI for STEMI at 1,204 hospitals in the Cath PCI Registry between 2007 and 2011	Outcomes of radial access for PCI in patients with ST-segment elevation myocardial infarction (STEMI)	The transradial approach was associated with lower bleeding rate and reduced in-hospital mortality
Feng K, et al. 2014 [25]	Retrospective cohort study	334 end-stage liver disease (ESLD) patients	Transfemoral and transradial cardiac catheterizations in ESLD patients	TR group had a significantly lower rate of pseudoaneurysms and bleeding complications
Jolly SS, et al. 2009 [31]	Meta-analysis	Randomized trials comparing radial versus femoral access coronary angiography from 2005 to April 2008 were included	The objective of this meta-analysis was to determine if radial access reduces major bleeding and as a result can reduce death and ischemic events compared to femoral access	Radial access reduced major bleeding and there was a corresponding trend for reduction in ischemic events compared to femoral access. Large randomized trials are needed to confirm the benefit of radial access on death and ischemic events
Neill J, et al. 2010 [34]	Clinical trial	Femoral access cases (n = 848, 412 diagnostic, 436 percutaneous coronary interventions [PCIs]) and radial access cases (n = 965, 459 diagnostic, 506 PCIs) were assessed	Fluoroscopy time (FT) and dose-area product (DAP) were recorded for all radial access and femoral access procedures during default femoral access, transition phase (femoral access and early radial access), and default radial access	Transition from femoral access to radial access for diagnostics and PCI increased FT. DAP increased for diagnostic radial access but not PCI compared with femoral access. FTs for radial access diagnostic cases decreased with experience
Kiemeneij F, et al. 1997 [39]	Clinical trial	A randomized comparison between transradial, trans-brachial and transfemoral PTCA with 6F guiding catheters was performed in 900 patients	Comparison of procedural and clinical outcomes of percutaneous transluminal coronary angioplasty (PTCA) performed with 6F guiding catheters introduced through the radial, brachial or femoral arteries	With experience, procedural and clinical outcomes of PTCA were similar for the three subgroups, but access failure is more common during TR - PTCA. Major access site complications were more frequently encountered after trans-brachial and TF - PTCA
Kołtowski L, et al. 2014 [40]	Randomized clinical trial	103 MI patients	Cost effectiveness and complication of Minor bleedings in transradial versus transfemoral access percutaneous coronary interventions for STEMI	The indirect costs were lower in the radial group. Transfemoral approach had a higher risk of access-related bleedings than transradial approach
Singh G, et al. 2016 [41]	Retrospective study	163 radial and 180 femoral access	Differences in the procedural variables between transradial and transfemoral access for coronary angiography, with cardiology fellows as the primary operators	Radial procedures were associated with more radiation and prolonged procedural time. Although total procedural time decreased for radial cases with the level of training, total radiation dose did not decrease
Iqa A, et al. 2014 [42]	Clinical trial	507 MI patients	To compare the clinical results of transradial and transfemoral in MI patients who had cardiogenic shock and underwent PCI	TR approach is associated with fewer major bleeding and vascular complications than TF approach especially in complicated cardiogenic shock patients
Brueck M, et al. 2009 [43]	Randomized clinical trial	1,024 MI patients	TR approach comparison with TF approach in the standard population of patients undergoing coronary catheterization	The rate of major vascular complications was negligible using the TR approach

## Conclusions

We conclude that although the transfemoral is being an old traditional approach, the transradial is the more modern approach. The most imperative question is that, are both of these approaches useful?. The evidence implies that both of these methods have been useful and fruitfully used in the past and the present. However, like any procedure, these two methods have their complications as well several studies suggest that the modern approach overweights in benefits with the comparison to the classical approach.

Complete transition from femoral approach to a radial approach is safe and successful in many cardiovascular procedures. The findings of the recent studies have shown that the transradial approach in cardiac catheterization is safe, cost-effective, and feasible with similar results to those of the transfemoral approach. However, duration and radiation exposure are higher in the transradial access. As the time passes, more research is being conducted and obviously more studies will be published in the future that will be able to target to find how to lessen the duration and the radiation exposure in transradial approach. Thus, the future studies will broaden our knowledge about the further possible benefits and complications of both the approaches.
